# Influence of hydrothermal synthesis parameters on the properties of hydroxyapatite nanoparticles

**DOI:** 10.3762/bjnano.7.153

**Published:** 2016-11-04

**Authors:** Sylwia Kuśnieruk, Jacek Wojnarowicz, Agnieszka Chodara, Tadeusz Chudoba, Stanislaw Gierlotka, Witold Lojkowski

**Affiliations:** 1 Laboratory of Nanostructures, Institute of High Pressure Physics (UNIPRESS), Polish Academy of Sciences, Sokolowska Street 29/37, 01-142 Warsaw, Poland

**Keywords:** hydroxyapatite, microwave hydrothermal synthesis, nanoparticle size control, physical properties of HAp NPs, room temperature synthesis

## Abstract

Hydroxyapatite (HAp) nanoparticles of tunable diameter were obtained by the precipitation method at room temperature and by microwave hydrothermal synthesis (MHS). The following parameters of the obtained nanostructured HAp were determined: pycnometric density, specific surface area, phase purity, lattice parameters, particle size, particle size distribution, water content, and structure. HAp nanoparticle morphology and structure were determined using scanning electron microscopy (SEM) and transmission electron microscopy (TEM). X-ray diffraction measurements confirmed crystalline HAp was synthesized, which was pure in terms of phase. It was shown that by changing the synthesis parameters, the diameter of HAp nanoparticles could be controlled. The average diameter of the HAp nanoparticles was determined by Scherrer’s equation via the Nanopowder XRD Processor Demo web application, which interprets the results of specific surface area and TEM measurements using the dark-field technique. The obtained nanoparticles with average particle diameter ranging from 8–39 nm were characterized by having homogeneous morphology with a needle shape and a narrow particle size distribution. Strong similarities were found when comparing the properties of some types of nanostructured hydroxyapatite with natural occurring apatite found in animal bones and teeth.

## Introduction

Hydroxyapatite (HAp) is a naturally occurring mineral (a form of calcium apatite), which is also an inorganic component of bones, with approximately 8 wt % water, 22 wt % protein and 70 wt % mineral. HAp is a form of calcium phosphate with the chemical formula Ca_10_(PO_4_)_6_(OH)_2_ and a hexagonal crystalline structure. Bones contain about 65 wt % hydroxyapatite, a needle-shaped compound with a width of 5–20 nm and length of 60 nm, which is responsible for rigidity and strength [1–2].

The wide range of potential applications of nanostructured HAp have drawn much attention among researchers, industry and investors. Hydroxyapatite is characterized by its biocompatibility and osteoconductivity. The material has been commonly and successfully used in regenerative medicine and in drug delivery systems [3–4]. Nanostructured hydroxyapatite particles can be applied as building blocks for damaged enamel in caries therapy [5]. Various studies demonstrate that owing to its high surface energy, nanoscale HAp can enhance its mechanical features, enabling a quicker implant surface turnover [6]. Hydroxyapatite materials are used as substitute bones, antiwrinkle creams, and sun creams. They are used in the production of toothpaste formulations and to speed up wound healing. There are patches and ointments using the unique properties of HAp that were developed by scientists in Poland [7]. Hydroxyapatite has also received great interest in the regeneration of animal bone loss.

It has been proved that HAp nanoparticles (nano-HAp) are better positioned to serve as an apatite substitute of bone in biomedical applications than micrometer-sized hydroxyapatite (micro-HAp) [8]. The impact of nano-HAp particles with different morphology on highly malignant melanoma cells was analyzed. The obtained results showed that proliferation of such malignant cells was inhibited more efficiently by the occurrence of the nanoscale effect than by HAp particle morphology [9]. Another study analyzed the effects of different sized nano-HAp – ranging from 20 to 80 nm – on the proliferation of bone-related cells (bone marrow mesenchyme stem cells and osteosarcoma cells) [10]. The cell culture experiment confirmed that in comparison to conventional HAp, cytophilicity of the nanophase mineral improved with nano-HAp. In addition, an increased viability and spread of stem cells was observed for nano-HAP, in particular for the smallest 20 nm particles. In the same vein, the smallest 20 nm particles turned out to be the best inhibitor of growth of osteosarcoma cells, yet all nano-HAp retarded the growth process. Another study also reported the antitumor action of nano-HAp [11].

It is believed that nano-HAp demonstrates desirable cell multiplication in order to optimize biological functionality, a property which is highly dependent on particle size [8]. The above mentioned findings play an important role in the comprehension of nano-HAp biological activity and cytophilicity during biomineralization.

Its ability to circulate in blood and its capacity to deliver a payload to cells and tissues makes nano-HAp a strong candidate to enhance the current effectiveness of disease diagnosis, which was already proved by applying nanoparticles to bioimaging and therapy [3,12].

HAp-based compounds and substances can also play a significant role in waste management and catalyst production [13–14] because of HAp’s high sorption activity to numerous ions including several heavy metals and radionuclides [15]. A model compound reflecting bone mineral phase is a nonstoichiometric hydroxyapatite with a molar ratio different than 1.67. Nevertheless, regarding biological apatite, the main substituent is carbonate, which normally occurs at 5 to 8 wt % in bone mineral. Moreover, calcium deficiency is always observed in biological apatites along with a relatively low degree of crystallinity – with diameter on the order of 0.2 µm and smaller [16–18].

In the hydroxyapatite structure, PO_4_^3−^ anions can be substituted to a certain extent by carbonate groups, whereas calcium ions can be substituted by magnesium (≈0.7 wt %), sodium (≈0.9 wt %), potassium (0.03 wt %) chlorine (0.13 wt %), fluorine (0.03 wt %) and a few trace elements: Sr, Pb, Zn, Cu, and Fe. The occurrence of the aforementioned elements affects the activity of bone-cell-related enzymes. The introduction of Mg^2+^ and CO_3_^2−^ ions causes a decrease in the size of crystals and an increase in solubility. The high reactivity of bone apatite is a result of low crystallinity. The reactivity is reflected in bone resorption processes. The amount of elements substituted depends on the conditions under which that structure is formed. Their occurrence also influences stoichiometry, crystallinity, and thermal and chemical stability of a compound [19]. In line with the increase in calcium deficiency, the number of crystalline structure defects, caused by calcium ion vacancies, increases, diminishing material stability, and as a result, enhancing its solubility [20–21].

A range of various methods have been developed in order to produce HAp powder, among others, combustion preparation [22], and numerous wet chemistry techniques, such as direct precipitation from aqueous solutions, electrochemical deposition [23], sol–gel processes [24] and hydrothermal synthesis [25–27] (Table 1).

**Table 1:** Summary of methods of obtaining HAp.

Ref.	Starting materials	Synthesis and process methods^a^	Particle shape/size	End products

[25]	(Ca(NO_3_)_2_·4H_2_O), (NH_4_)_2_HPO_4_	ppt + 48/72 h in 200 °C + ultrasonic bath washing + drying	median length 100–600 nm, median width 20–40 nm	monetite CaHPO_4_ after drying
[28]	(Ca(NO_3_)_2_)·4H_2_O, H_3_PO_4_, NH_4_OH	ppt + MW + washing + calcination (500 °C)	nano-rods *L* = 37 nm, *D* = 8 nm	NH_4_NO_3_ before washing
[29]	(Ca(NO_3_)_2_)·4H_2_O, (NH_4_)_2_HPO_4_	ppt + centrifugation (6000 rpm for 10 min) + washing + freeze drying + calcination (550 °C/5 h)	rod-like crystals *L* = 40 nm, *D* = 15 nm	NH_4_NO_3_ before washing
[30]	Ca(OH)_2_ calcinate to form CaO (improves the reactivity of lime), H_3_PO_4_	ppt (20 °C) + 24 h ripening + washing +calcination (900 °C)	300 nm	no impurities because of washing
[31]	CaCl_2_, KOH, KH_2_PO_4_	ppt (70 °C) 1 h + reflux time (1 week) + filtration + washing + drying	length of the crystals 200–400 nm; SSA 70 m^2^/g	Cl^−^ before washing, K^+^ could substitute calcium ions into the HAp crystal lattice
[32]	CaSO_4_·0.15H_2_O, (NH_4_)_2_HPO_4_	ppt (25 °C) + 21 days in 25 °C + washing + drying	crystal size of 1 to 8 μm	(NH_4_)_2_SO_4_, H_2_SO_4_ before washing, brushite after drying

^a^ppt, precipation; MW, microwave.

In the majority of cases, the synthesized powder is a stoichiometric hydroxyapatite, and oftentimes contains additional phases and substances such as by-products or unreacted substrates [25,32]. Apart from all aforementioned methods, the hydrothermal technique seems to have many advantages [33]. Microwave hydrothermal synthesis (MHS) is a development of the hydrothermal technique [34]. MHS can be defined as a microwave-assisted process in a closed reaction vessel, inducing decomposition or a chemical reaction(s) between the precursor(s) in the presence of a water solvent at a temperature higher than the water boiling point. The technique was developed early on in order to synthesize HAp and was well received because it is easy to use, cost efficient and environmentally friendly [35].

In a previous study [26–27], we reported microwave-driven hydrothermal synthesis of nano-HAp, with average particle size ranging from 6 to 30 nm, which varied with synthesis time. Further, it was found that the Ca/P ratio of the NPs was synthesis-time dependent. The purpose of this study was to further develop the previously reported synthesis methods in order to: further extend the range of particle sizes, scale up the synthesis process by six orders of magnitude of six types of nano-hydroxyapatite, and to demonstrate that a precise control of the average particle size and particle size distribution can be maintained and these parameters can still be precisely tuned.

## Experimental

### Materials

The HAp nanopowder was synthesized via a simple precipitation method (exactly in the acid/base neutralization process). The precursors for the synthesis were calcium hydroxide (10.3458 g) (Ca(OH)_2_, pure, CHEMPUR, Poland) and orthophosphoric acid (5.7 mL) (H_3_PO_4_, 85 wt % solution, analytically pure, CHEMPUR, Poland). Deionized water was used for the synthesis (450 mL) (HLP 20UV, Hydrolab, Poland). The molar ratio of the starting materials used was Ca/P 1.67. Phosphoric acid was added dropwise to the water suspension of calcium hydroxide at a rate of 0.01 mL every 0.5 s (Titrator, SI Analytics, Titronic universal, TZ3260, Germany); hydroxyapatite was formed with the following reaction scheme:

[1]
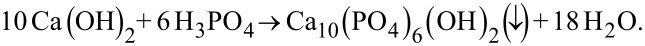


**Figure 1:**
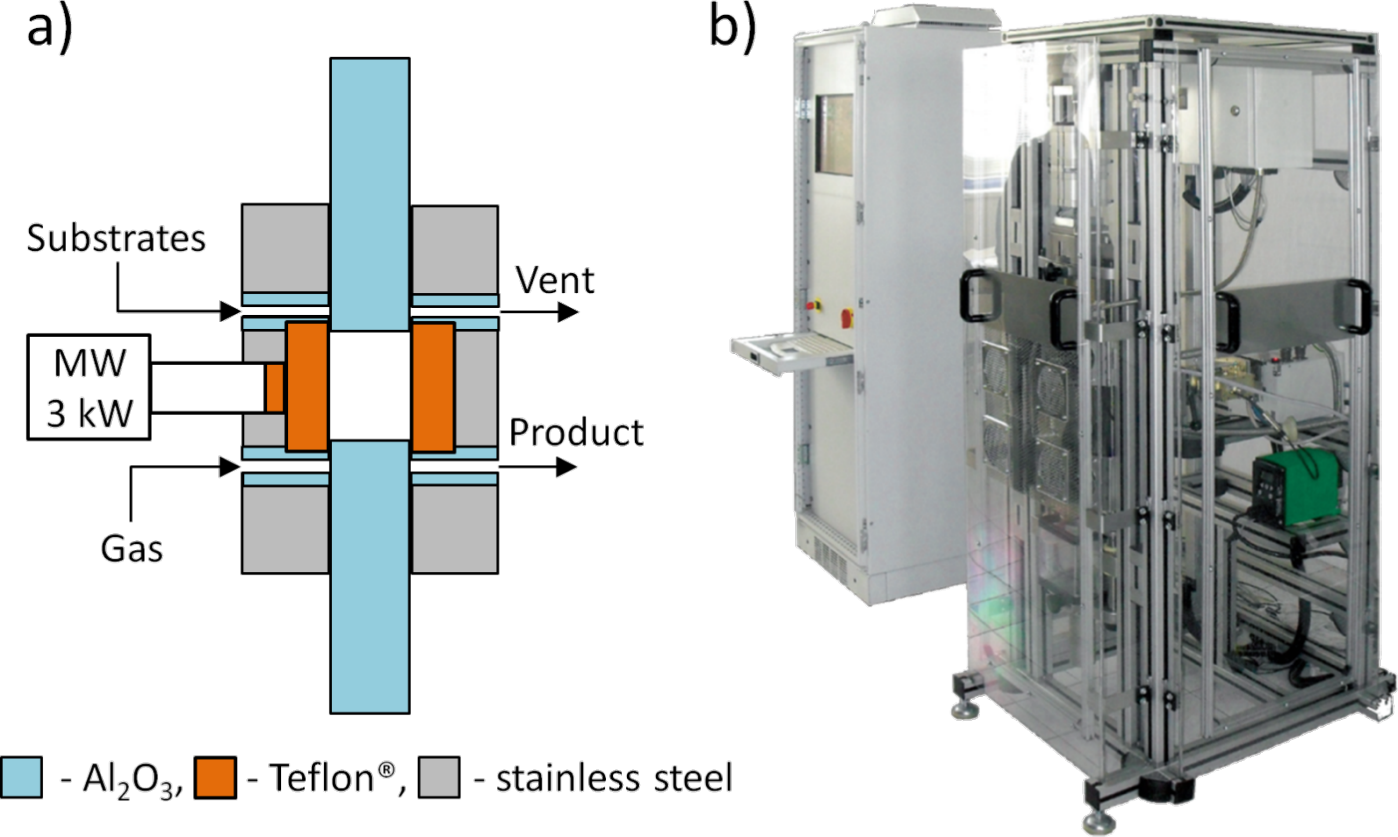
The MSS2 reactor [36]: a) general draft of the MSS2 reactor; b) view of the prototype; c) principle of operation of the load and unload system.

The hydroxyapatite precipitate was intensively stirred using a mechanical stirrer at room temperature. The suspension of Type 1 hydroxyapatite was obtained after 30 min of additional stirring. In order to obtain Type 2–Type 6 hydroxyapatite materials, the reaction water suspension was transferred into a capped Teflon^®^ vessel and heated using microwave radiation in the MSS2 reactor (ITeE-PIB, IHPP PAS, Ertec Poland [36]) as shown in Figure 1.

The parameters of the reactor are as follows: *p* <= 6 MPa, *T* <= 260 °C, *V* = 470 mL, *P* = 3 kW. Hydroxyapatite (Type 2–Type 6) was obtained by microwave hydrothermal synthesis (MHS). The MSS2 reactor (Figure 1) permits rapid, uniform heating and synthesis under high purity conditions in a closed vessel with precise control of the reaction time (Table 2).

**Table 2:** Synthesis parameters.

Sample	Synthesis parameters of the MSS2 reactor			

	Time (s)	Pressure (Bar)	Temperature (°C)	Power (kW)

HAp Type 1	obtained without using the reactor			
HAp Type 2	55	≈1	115	3
HAp Type 3	90	3	140	3
HAp Type 4	600	3	140	3
HAp Type 5	600	10	190	3
HAp Type 6	1200	20	220	3

The synthesis was carried out in the MSS2 reactor at a constant power during the process, over the duration times of 55, 90, 600 and 1200 s, depending on the type of the synthesized powder (Type 2–Type 6) at varied pressures (Table 2).

The only by-product of hydroxyapatite synthesis is water, which enables the washing of synthesis products to be skipped. The as-obtained precipitate was dried in a lyophilizing cabinet (Lyovac GT-2, SRK Systemtechnik GmbH, Germany), attached with a vacuum pump by Leybold Trivac. The drying parameters depended on the amount and type of powder batch.

### Methods

**X-ray diffraction (XRD).** XRD patterns of nano-HAp powders were collected on an X’Pert PRO, PANalytical diffractometer equipped with a copper anode (Cu Kα_1_) and an ultra-fast PIXcel^1D^ detector [37]. The analysis was performed at room temperature in the range 2θ = 10–100° with a step of 0.03°. The average diameter of nano-HAp crystallites (*d*_hkl_) was calculated on the basis of the Scherrer’s equation [38].

**Density measurements**. The density of two nano-HAp types were measured using a helium pycnometer (AccuPyc II 1340, Micromeritics) at 24 ± 2 °C, according to ISO 12154:2014.

**Specific surface area (SSA).** SSA of the nanopowders was measured by analysis of the BET isotherm method using a Gemini 2360 instrument by Micromeritics according to ISO 9277:2010. Before the density and SSA measurements were carried out, the powders were dried at 150 °C for 2 h in a constant flow of helium (FlowPrep 060 desorption station by Micromeritics). The average diameter of the particles (*d*_BET_) was calculated on the basis of SSA and density (see Equation 2); assuming that all HAp particles were spherical and identical.

[2]
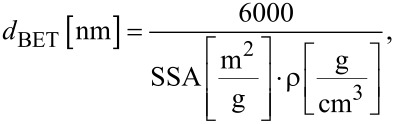


where *d*_BET_ is the average particle diameter calculated using the SSA value, SSA is the specific surface area calculated using the BET isotherm, and ρ is the material density.

**TG-DSC.** TG-DSC analysis was carried out using an STA 449 F1 Jupiter by Netzsch. The analysis was performed with a heating rate of 5 °C/min; the top temperature was 1350 °C. The measurement was performed under a constant flow of helium at 60 mL/min.

**SEM and TEM.** The morphology of the nanopowder samples was examined using SEM (Ultra Plus; Carl Zeiss Meditec AG, Jena, Germany) and transmission electron microscopy (TEM) (JEM2000EX; JEOL, Tokyo, Japan). The TEM, high-resolution TEM, and selected area electron diffraction tests were conducted at 200 kV. The samples for the TEM observations were prepared by dropping the methanol particle dispersion, created by an ultrasonic technique, on a carbon film supported on a 300-mesh copper grid. Additionally, TEM tests were used to determine the nanoparticle size distribution. The particle size histograms were obtained by examining an area of a sample containing about 250 nanoparticles. The nanoparticle shape was nearly to spherical. The obtained histograms were fitted to either normal or log-normal distributions (Χ-squared test and Pearson’s coefficient) [26].

**Chemical composition of the powders.** The experimentally measured ion content in the powders may not be identical to that in the solutions. The chemical composition analysis of powders was examined by inductively coupled plasma optical emission spectrometry (ICP-OES) with induction in argon plasma (Thermo Scientific, iCAP 6000 series, United Kingdom). The samples analyzed using ICP-OES were prepared as follows: 5 mg of powder was weighed in a 110 mL Teflon^®^ vessel and 15 mL of deionized water (HLP 20UV, Hydrolab, Poland) was added. Then, 6 mL of HNO_3_ was added and the solution was subjected to one microwave heating cycle in the microwave reactor (Magnum II, Ertec, Poland). After cooling, the sample volume was replenished to 50 mL with deionized water [39].

**Crystallite size distribution.** A Nanopowder XRD Processor Demo was used for the analysis of the XRD peak profile by using an analytical formula for polydispersive powders [40]. This technique provides four parameters: average particle size, error of average particle size, dispersion of size and error of dispersion of size. Hence, a full particle size distribution curve and an estimation of “thickness” of this curve (error bars) are obtained. For calculating the crystallite diameter and size distribution, the Nanopowder XRD Processor Demo web application employs equations derived for spherical crystallites. The website http://www.science24.com/xrd provides an on-line tool where diffraction files can be directly dropped. The files are processed on a server to extract particle size distribution for XRD peaks. Unlike standard fitting, the tool does not act in the reciprocal space at but solves all sets of equations in a few auxiliary spaces simultaneously. This allows an analysis of XRD data with heavily convoluted reciprocal space peaks [41].

## Results and Discussion

The precise control of particle size and particle size distribution is one of the main challenges faced by nanoparticle technology [42]. An increased scale up of hydroxyapatite production was achieved as a result of increasing the initial volume of reagents and using a new reactor, MSS2, as described previously. The precursor, and at the same time HAp Type 1, was synthesized, obtaining ≈500 mL of suspension. This was dried in a lyophilizing cabinet and ≈14 g (95% efficiency) was obtained from a single synthesis. In previous work [26–27] the precursor was prepared from 75 mL of the suspension (where HAp was not obtained at room temperature). 350 mL of the suspension (≈11 g of HAp) was fed to the MSS2 reactor (maximum permitted feedstock volume in MSS2 reactor’s reaction chamber), in comparison with previous work [26–27], where a different microwave reactor was used (Ertec Magnum-II) and only 70 mL of the suspension (≈2 g of HAp) could be synthesized at one time. When using the MSS2 reactor, up to 150 g of HAp could be synthesized over one day (10 working hours), while in the case when using the Ertec Magnum-II reactor, up to 20 g could be produced [26–27].

**Figure 2:**
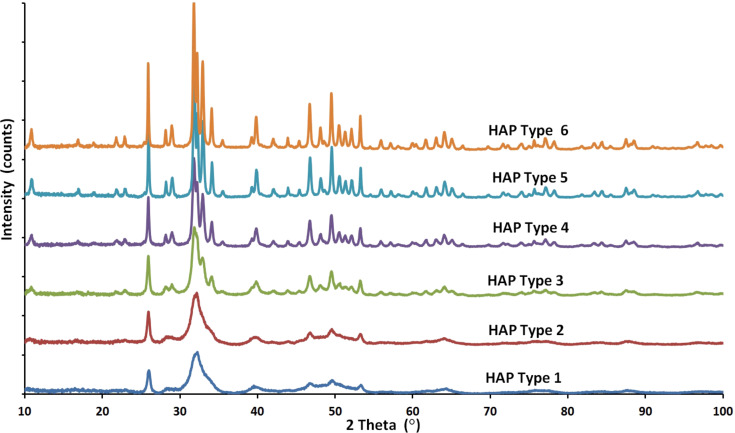
XRD patterns of HAp powders.

An XRD analysis of all obtained HAp powders was carried out. The results confirmed that Type 1–Type 6 HAp powders were pure crystalline products (Figure 2).

**Figure 3:**
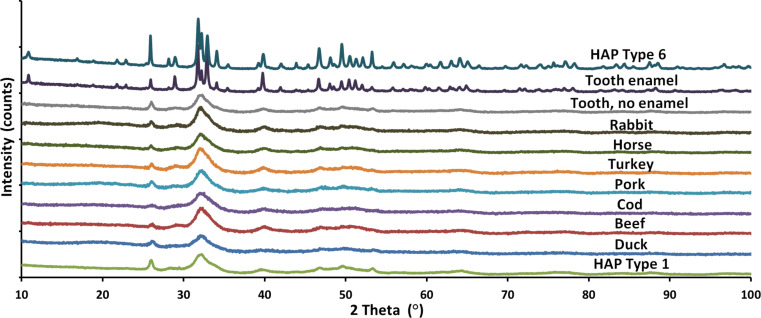
XRD patterns of a duck bone, a beef bone, a pork bone, a turkey bone, a horse bone, a rabbit bone, a cod bone and a tooth with and without enamel, as well as HAp Type 1 and Type 6 nanopowders.

Based on the diffraction patterns, the gradual growth of the crystallites (nanoparticles) from Type 1 to Type 6 was observed. The presence of other crystalline phases or any amorphous components was not observed. In addition, the XRD method was used to test a beef bone, a rabbit bone, a horse bone, a turkey bone, a pork bone and a duck bone (Figure 3).

Additionally, the XRD results of a cod bone, a human tooth with enamel and a tooth root were summarized. Pastilles with a diameter of 12 mm, obtained by pressing pulverized bones under a pressure of 10 MPa, were used for the EDX measurement. The above mentioned natural samples were used as a comparison and reference to natural apatite. The crystal structures of the tested samples of bones and the tooth without enamel are almost identical. The diffraction pattern of HAp Type 1 nanopowder coincides with the result of HAp NPs for the tooth without enamel. It is also very similar to bone hydroxyapatite NPs. The publication by Martin et al. [43] contains XRD results of human bone. When juxtaposing the XRD results of human bone and of a HAp Type 1 sample, it can be found that they look identical. The HAp particles obtained and described in this paper display peaks with hexagonal crystalline structure and P63/m space group, which was confirmed by TEM results. The presence of no other crystalline phase than hydroxyapatite was detected. The peaks characteristic of hydroxyapatite were observed at angles of 26° and 30–34° [44]. These results for HAp Type 1 confirm that the obtained hydroxyapatite is pure and homogeneous. For HAp Type 1 and natural apatites juxtaposed in this paper and juxtaposed with the results for human bone [43], the peaks are located in the same places in diffraction patterns and are broadened at the same peak proportions. The peak broadening for HAp Type 1 and natural HAp of animal bones and human bone displays the same crystalline morphology, which means that synthetic HAp Type 1 is characterized by the identical morphology to human HAp [43], and identical to HAp occurring in animal bones (Figure 3). On this basis it can be stated that HAp Type 1 is a synthetic equivalent of natural bone. HAp Type 6 is characterized by a larger nanoparticle size, which is visible through a narrower width of the peaks in the diffraction pattern. The crystalline structure of HAp Type 6 is very similar to the crystalline structure of tooth enamel.

Type 1–Type 6 hydroxyapatite indicates high nonstoichiometry (Table 3): the Ca/P molar ratio is 1.61 ± 0.03, where synthesized hydroxyapatites are apatites with calcium deficiency (Ca-def). The molar ratio of the obtained hydroxyapatites is very similar to the Ca/P molar ratio present in natural bone (1.65), or enamel (1.62) [5,45]. The Ca/P molar ratio does not depend on the synthesis parameters.

**Table 3:** The Ca/P ratio determined by ICP-OES measurements.

Sample	Ca/P

HAp Type 1	1.64 ± 0.02
HAp Type 2	1.61 ± 0.02
HAp Type 3	1.61 ± 0.02
HAp Type 4	1.59 ± 0.01
HAp Type 5	1.61 ± 0.02
HAp Type 6	1.60 ± 0.03

The papers by Smolen et al. [26–27] are very interesting publications, describing that the Ca/P molar ratio for the obtained HAp powders depended on the duration and temperature of the synthesis reaction carried out in an Ertec Magnum II microwave reactor. They adopted a constant molar ratio of reagents of Ca/P 1.67 for all syntheses. In order to prepare the reaction suspension, H_3_PO_4_ was added dropwise to the water suspension of Ca(OH)_2_ at a speed of 0.01 mL every 3 s, while intensely stirring the suspension at room temperature. The authors did not obtain HAp NPs at room temperature. They treated the suspension obtained at room temperature solely as the precursor for microwave hydrothermal synthesis of HAp NPs. After the completed syntheses they flushed the obtained suspensions with ethanol and water and subsequently dried in a laboratory drying cabinet [26–27].

The synthesis described herein reports a similar technology for obtaining HAp NPs. We used the same reagents (H_3_PO_4_, Ca(OH)_2_) and we adopted the same constant molar ratio of the reagents being Ca/P 1.67. However, our method differs in several very important aspects from the afore mentioned paper. Namely, we added H_3_PO_4_ dropwise to the Ca(OH)_2_ suspension at a six times greater speed (0.01 mL every 0.5 s). We did not flush the obtained suspensions but only dried them in the lyophilizing cabinet. At room temperature we always obtained fully crystalline HAp Type 1. In order to obtain HAp Type 2–6, we used a MSS2 microwave reactor, where HAp Type 1 suspension was used as the precursor. During our experiment, we did not observe any impact of synthesis duration and temperature on the Ca/P molar ratio of HAp powders. In order to confirm this, it was decided to synthesize a powder with a different starting Ca/P molar ratio and determine the parameters of the obtained powder. The hydroxyapatite precursor was prepared with calcium deficiency, with the molar ratio Ca/P 1.57. The synthesis procedure was the same as described in the section Materials. The results of ICP-OES tests revealed that powders (Type 1–Type 6) characterized by high nonstoichiometry were obtained with calcium deficiency, as was assumed. The molar ratio of the synthesized hydroxyapatite types was Ca/P 1.52 ± 0.01. The achieved results clearly show the manner in which stoichiometry was controlled. The stoichiometry was controlled by modifying the precursor at the initial synthesis stage (see the section Materials). Contrary to the previous results, the Ca/P ratio was not a function of the synthesis parameters [26–27].

We believe that the major reasons for differences between our method and the method presented by Smolen et al. [26–27] in terms of controlling the Ca/P molar ratio of HAp NPs are as follows: the use a different type of precursor, a different manner of flushing, and a different manner of drying.

The density of the HAp Type 1–Type 6 nanopowders was 2.86 to 3.03 g/cm^3^ (Table 4). Literature data show that the density of hydroxyapatite nanopowders is 3.05 g/cm^3^ [45]. For Type 1–Type 3 powders, the density values were lower by 4–6%. Such a difference results from the nano-size of the particles of the obtained nanopowder. In the case of nanomaterials, a decrease in density is observed in comparison with micropowders, because the material surface layer is not as closely packed as the whole volume of particles [46] (with the observed appropriately small surface in relation to the higher density value), or the presence of small quantities of the amorphous phase. The specific surface area ranged from 51 to 258 m^2^/g (Table 4), where the average particle diameter calculated based on the SSA was 8 to 39 nm (Table 5).

**Table 4:** Density and specific surface area (SSA) of synthesized hydroxyapatite powders.

Sample	SSA *a*_s_ ± σ (m^2^/g)	Density ρ_s_ ± σ (g/cm^3^)

HAp Type 1	258 ± 1	2.86 ± 0.02
HAp Type 2	211 ± 1	2.92 ± 0.02
HAp Type 3	149 ± 1	2.95 ± 0.01
HAp Type 4	85 ± 1	3.00 ± 0.01
HAp Type 5	61 ± 1	3.03 ± 0.01
HAp Type 6	51 ± 1	3.04 ± 0.01

**Table 5:** Comparison of particle diameter for the various HAp nanopowders, calculated by four different methods.

Sample	Average particle diameter from SSA/BET *d* ± σ (nm)	Average crystallite size from the Scherrer's equation		Average particle diameter from TEM *x*_c_ ± σ (nm)	Average crystallite size from Nanopowder XRD Processor Demo *d* ± σ (nm)

		Length (nm)	Width (nm)		

HAp Type 1	8.1 ± 0.1	19 ± 9	6 ± 2	6.5	9.6 ± 7
HAp Type 2	9.7 ± 0.1	24 ± 9	7 ± 0.5	7.3 ± 0.3	11.4 ± 6.4
HAp Type 3	13.7 ± 0.1	28 ± 12	14 ± 6	11.7 ± 0.3	15.7 ± 9.8
HAp Type 4	23.5 ± 0.3	38 ± 17	23 ± 6	18.4 ± 0.6	24.4 ± 16.9
HAp Type 5	32.5 ± 0.4	50 ± 20	30 ± 9	26.9 ± 0.6	38.4 ± 28.1
HAp Type 6	38.7 ± 0.6	60 ± 20	33 ± 9	34.8 ± 0.9	63.7 ± 45

**Figure 4:**
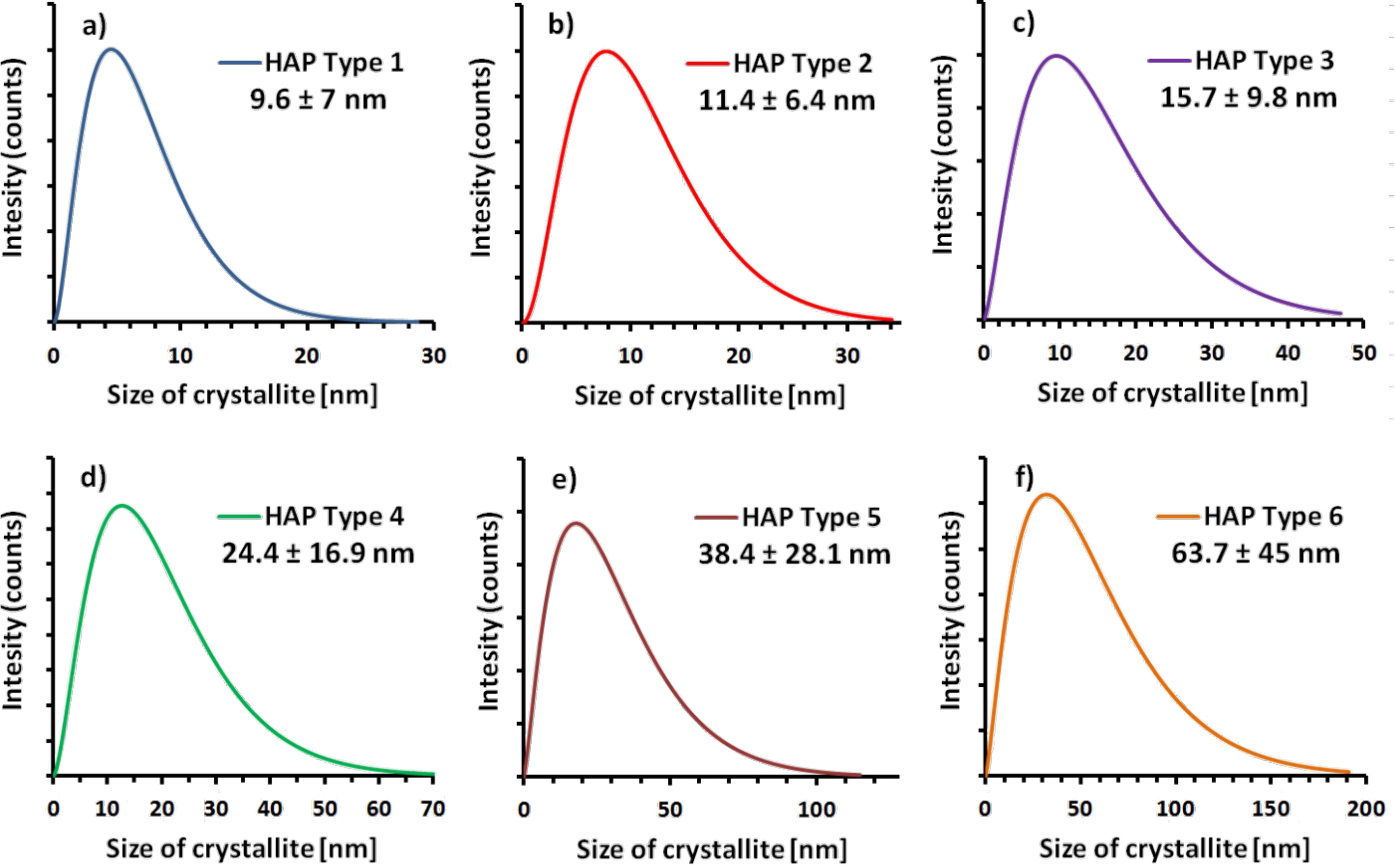
Crystallite size distribution, obtained using Nanopowder XRD Processor Demo [48]: a) HAp Type 1; b) HAp Type 2; c) HAp Type 3; d) HAp Type 4; e) HAp Type 5; f) HAp Type 6.

Based on an analysis of the XRD diffraction patterns, a distribution of crystallite sizes was determined for HAp NPs (Figure 4).

Depending on the synthesis time and applied pressure, the HAp nanomaterial density gradually increased and the SSA considerably decreased – the data are presented in Table 4. For Type 6 hydroxyapatite, a SSA of 51 m^2^/g and a density of 3.04 g/cm^3^ were found. The average particle diameter was calculated based on SSA and density results. For Type 1 hydroxyapatite, the average particle diameter was 8 nm, while for Type 6 it was 39 nm. For Type 3, a decrease in SSA to 149 m^2^/g was observed. The density increased by 0.09 g/cm^3^ to 2.95 g/cm^3^; the average particle size was 14 nm. Due to the longer synthesis time and accordingly higher temperature in the reactor, the SSA was reduced to 85 m^2^/g for Type 4, to 61 m^2^/g for Type 5, and to 51 m^2^/g for Type 6. Therefore, the density was found to be 3.00 g/cm^3^ (Type 4), while the particle diameter increased to 24 nm. For Type 6, the average diameter of the obtained particles was 39 nm and the density was 3.04 g/cm^3^, which was nearly equal to the literature value for hydroxyapatite [47].

The material was characterized by an average particle diameter between 10 and 64 nm from data obtained using Nanopowder XRD Processor Demo (Table 5). The calculations of the average crystallite size (XRD) coincide with an accuracy of 2–25 nm with average particle diameters calculated based on the SSA, which confirms that the nanopowder is monocrystalline and does not form aggregates. However, a change in the nanoparticle diameter is directly proportional to the density and inversely proportional to the SSA.

Hydroxyapatite is the primary component of bones and teeth [5]. The crystallite (particle) size of natural apatites present in bones (Table 6), calculated based on XRD using Scherrer’s equation, revealed a strong similarity to the results of synthetic Type 1 and Type 2 hydroxyapatites (Table 5).

**Table 6:** Comparison of crystallite sizes in bones as examples of natural apatite, calculated using XRD and Scherrer’s equation.

Examples of natural apatite	Average crystallite size from Scherrer's equation	

	Length (nm)	Width (nm)

Duck bone	17 ± 2	7 ± 1
Beef bone	21 ± 5	6 ± 1
Cod bone	19 ± 2	5 ± 1
Pork bone	16 ± 6	6 ± 1
Turkey bone	20 ± 7	6 ± 3
Horse bone	20 ± 2	8 ± 1
Rabbit bone	20 ± 3	6 ± 2
Tooth, no enamel	21 ± 4	8 ± 2
Tooth, with enamel	48 ± 30	40 ± 22

Thus, the hydroxyapatite that was synthesized displayed a structural similarity to bones and teeth. The width and length of crystallites in bones and teeth without enamel are comparable, where the values fall within the distribution range. Bones contain a considerable quantity of carbonate ions (5–8%). Apatite present in bones contains very few hydroxyl groups. Among the examples of natural apatites given, only the apatite present in tooth enamel contains less carbonates and a higher concentration of hydroxyl ions [49]. In her work, Sharon Kehoe [50] draws attention to the importance of atmospheric environment. She states that when chemical precipitating process occurs, adsorption of atmospheric CO_2_ can follow in a way that the carbonate anion in the form of CO_3_^2−^ can become imbedded into the crystalline lattice of HAp. This could give rise to the appearance of microscale stresses and deformations in the crystal lattice of the stoichiometric HAp [51]. Crystallographic changes in the structure of biological apatites are caused by carbonate substitutions in the apatite crystalline lattice [52]. Carbonate substitutions in the hydroxyapatite lattice are unstable and depend on synthesis temperature. Table 7 summarizes the results of the lattice parameters of synthesized HAp.

**Table 7:** Results of the lattice parameters determined by the XRD analysis for synthesized hydroxyapatite.

Sample	Lattice parameter *a* (Å)	Lattice parameter *c* (Å)	*a*/*c*

HAp Type 1	9.436 ± 0.003	6.874 ± 0.002	1.373
HAp Type 2	9.431 ± 0.002	6.878 ± 0.001	1.371
HAp Type 3	9.421 ± 0.001	6.878 ± 0.001	1.370
HAp Type 4	9.421 ± 0.001	6.878 ± 0.001	1.370
HAp Type 5	9.421 ± 0.001	6.877 ± 0.001	1.370
HAp Type 6	9.420 ± 0.001	6.877 ± 0.001	1.370

The highest value of the *a* lattice parameter was found for Type 1 HAp. Type 1 hydroxyapatite was obtained by a precipitation method at room temperature, where the quantity of CO_2_ dissolved in water is the greatest (increase in *a* direction), and lower when HAp was synthesized using the reactor (i.e., Type 2–Type 6, increased reaction temperature). An advantage of the product synthesized at room temperature is the greater carbonate content, which better correlates to the natural apatite present in bones.

Changes in the *a*/*c* ratio would translate into corresponding changes in the lattice parameters. Hence, lowering the *a*/*c* ratio would mean a reduction in the *a* lattice parameter and a simultaneous increase in the *c* lattice parameter, making it more beneficial than an increased *a*/*c* ratio, which efficiently reduces the crystallinity response [50]. The increased *a*/*c* ratio (an increase in *a* and a decrease in *c*) contributes to reduced crystallinity and the presence of a foreign phase, β-TCP [53].

When comparing the various methods of converting the XRD results, Scherrer’s equation and the Nanopowder XRD Processor Demo (Tab. 5) showed similar results that fell within the standard deviation of methods. The results of the XRD method calculated using Scherrer’s equation coincide with an accuracy of ≈5% with the results of the method based on SSA and density results. The average particle diameter of HAp samples obtained from TEM measurements (Table 5) is inconsistent with the results found by the other methods. These differences may be caused by the small quantity of the tested sample in comparison with the diffraction method, as well as sample image overlapping and particle shape.

**Figure 5:**
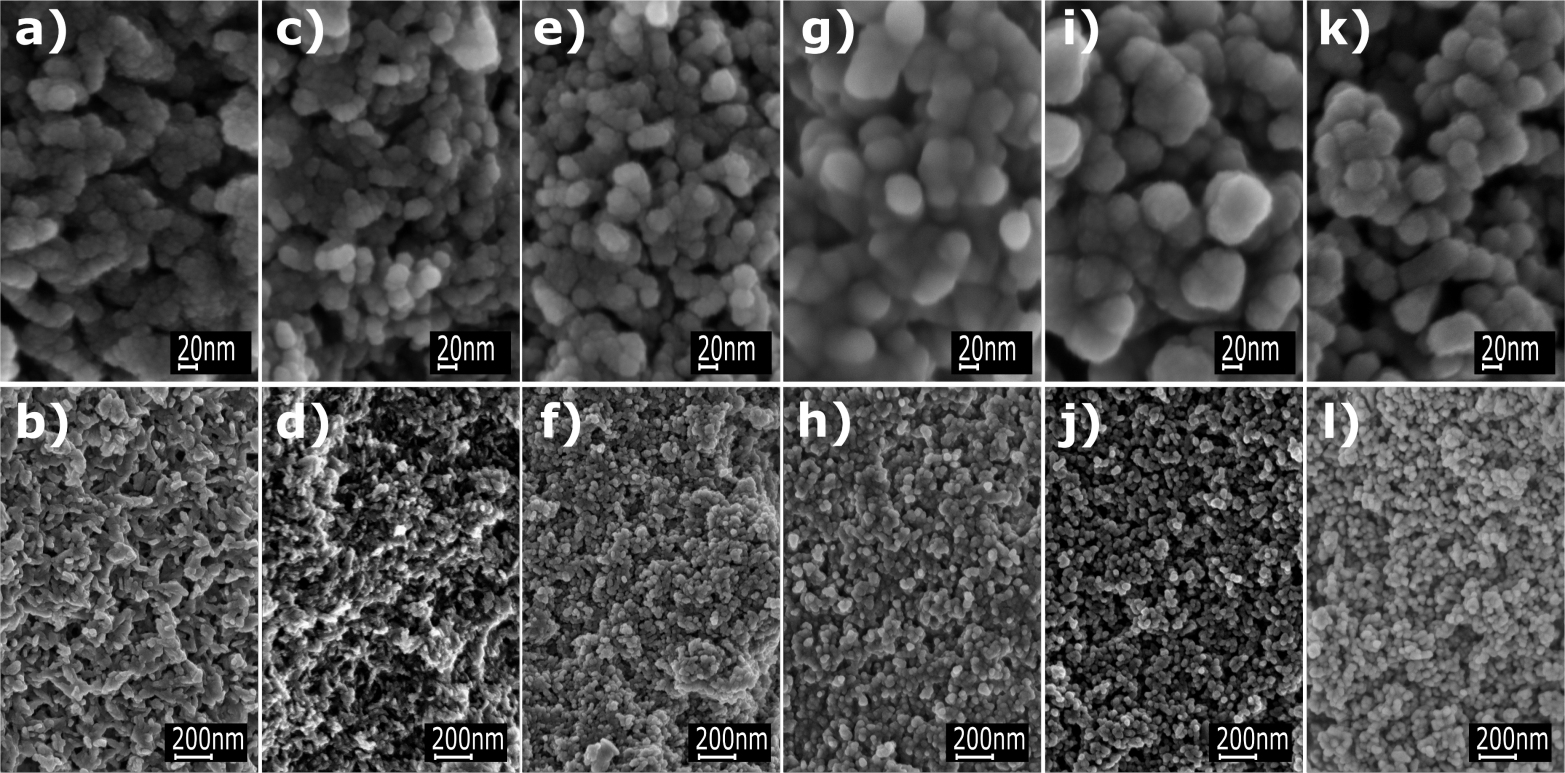
SEM micrographs of HAp powders: (a, b) Type 1; (c, d) Type 2; (e, f) Type 3; (g, h) Type 4; (i, j) Type 5; (k, l) Type 6.

**Figure 6:**
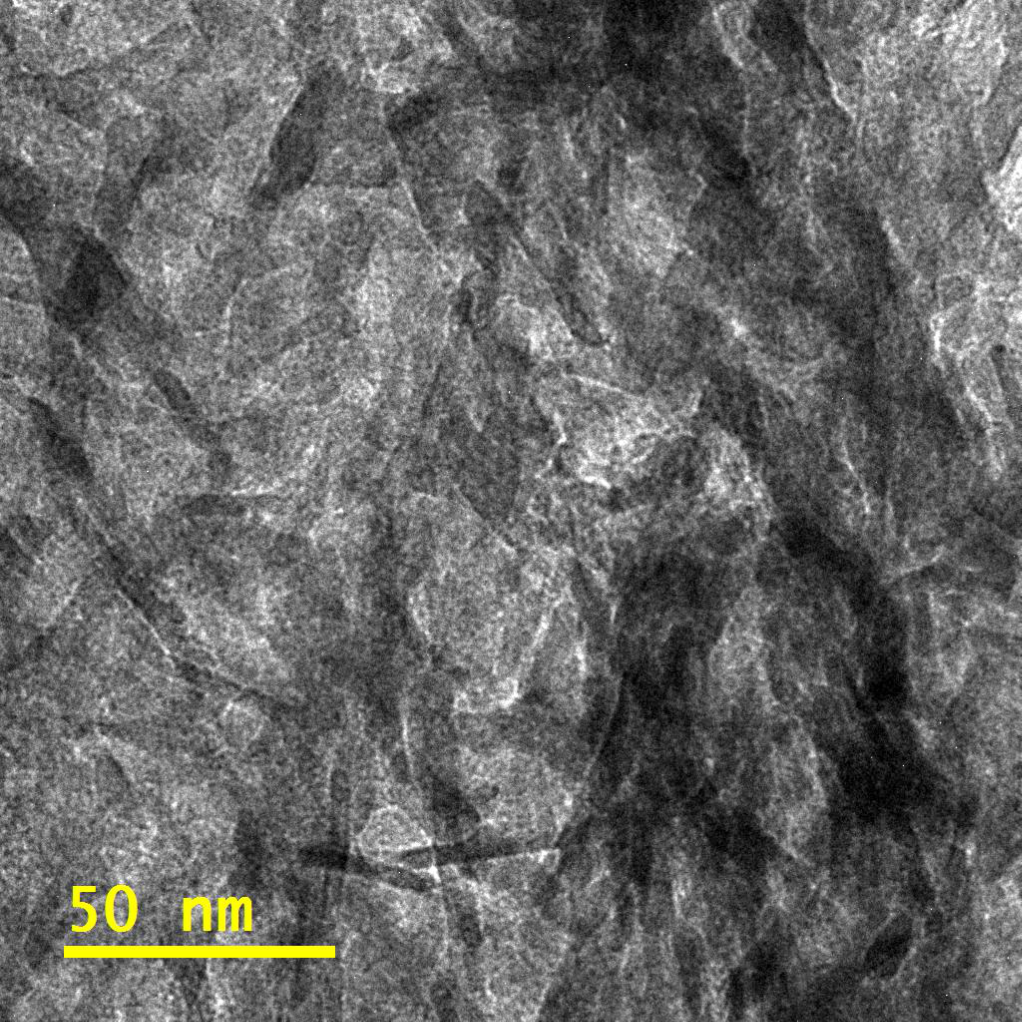
The bright field TEM image of Type 1 HAp.

**Figure 7:**
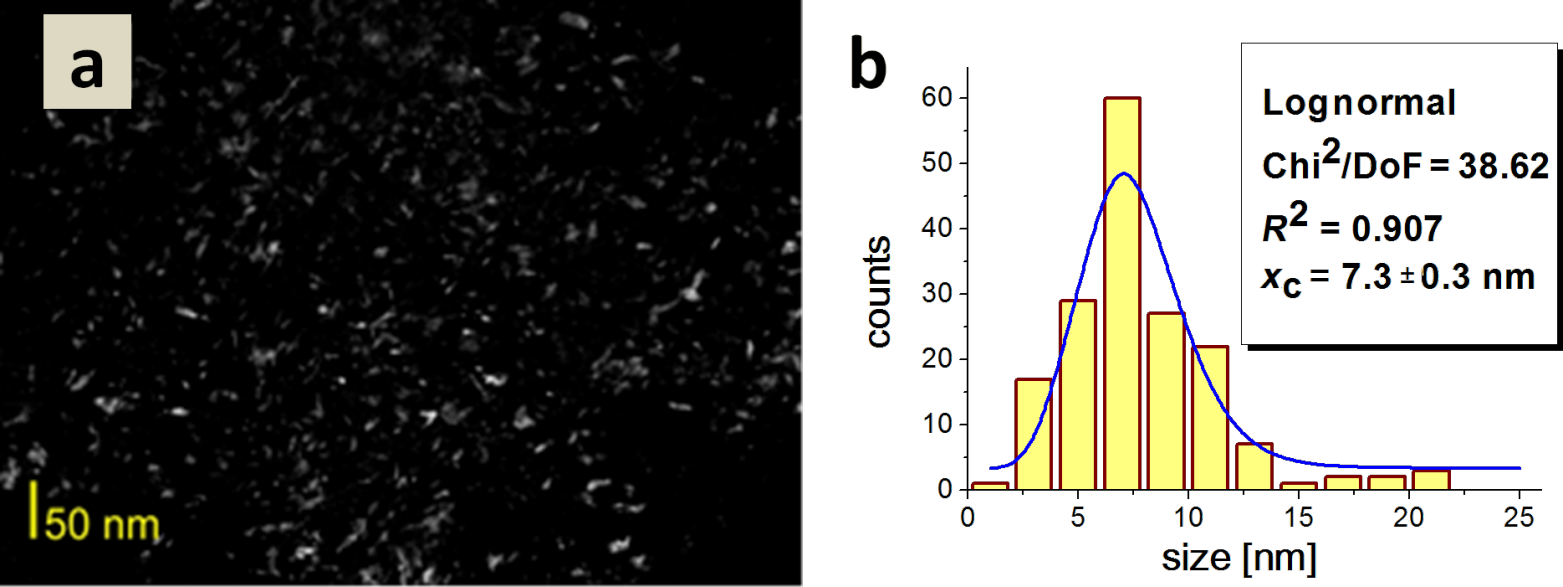
a) A dark field TEM image of Type 2 HAp; b) a histogram of the particle size distribution.

**Figure 8:**
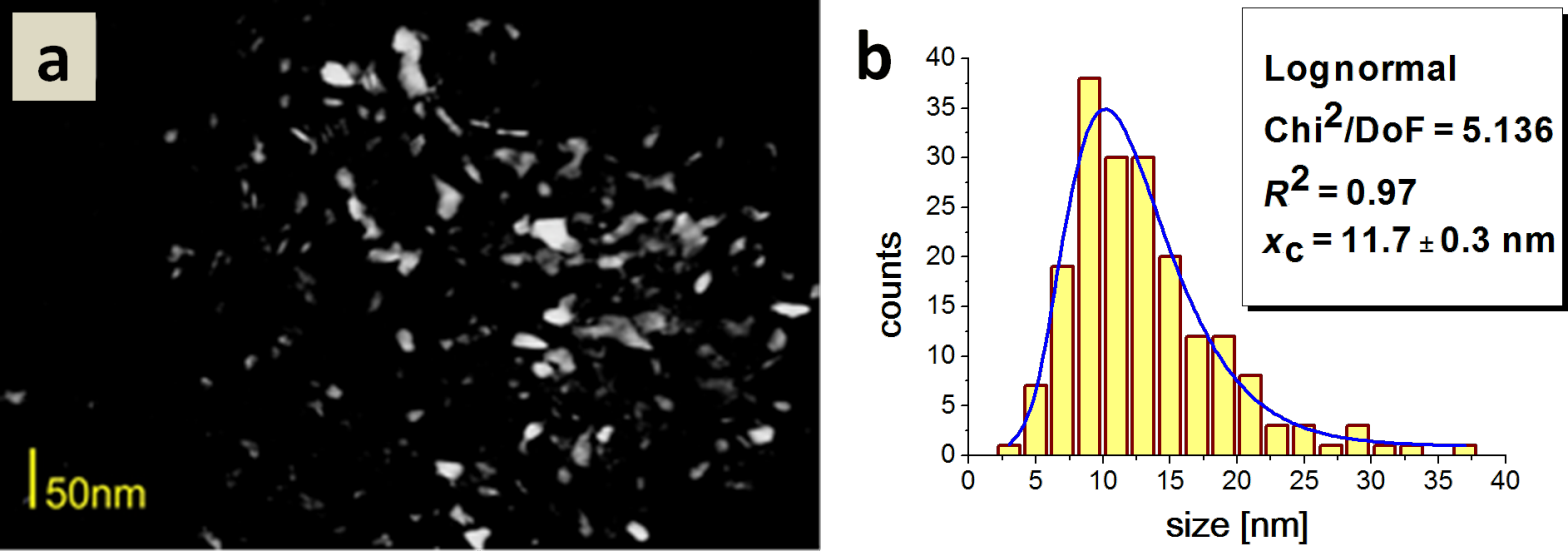
a) A dark field TEM image of Type 3 HAp; b) a histogram of the particle size distribution.

**Figure 9:**
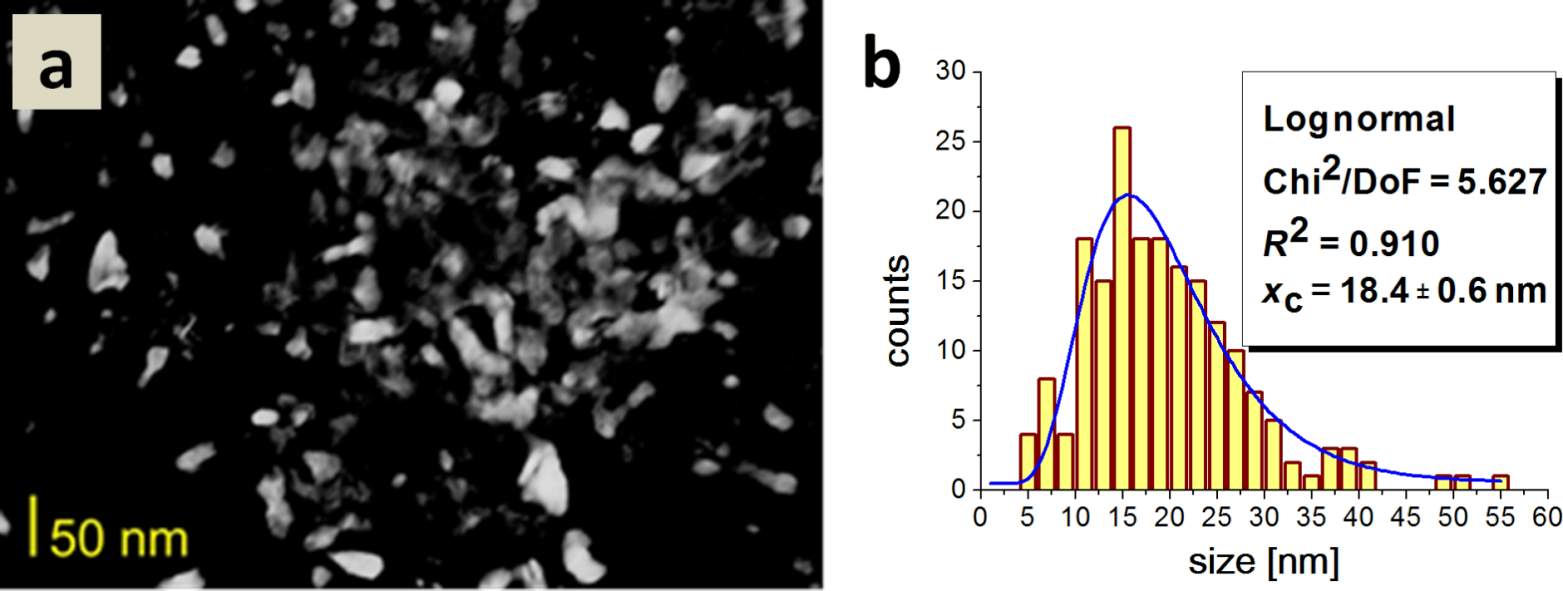
a) A dark field TEM image of Type 4 HAp; b) a histogram of the particle size distribution.

**Figure 10:**
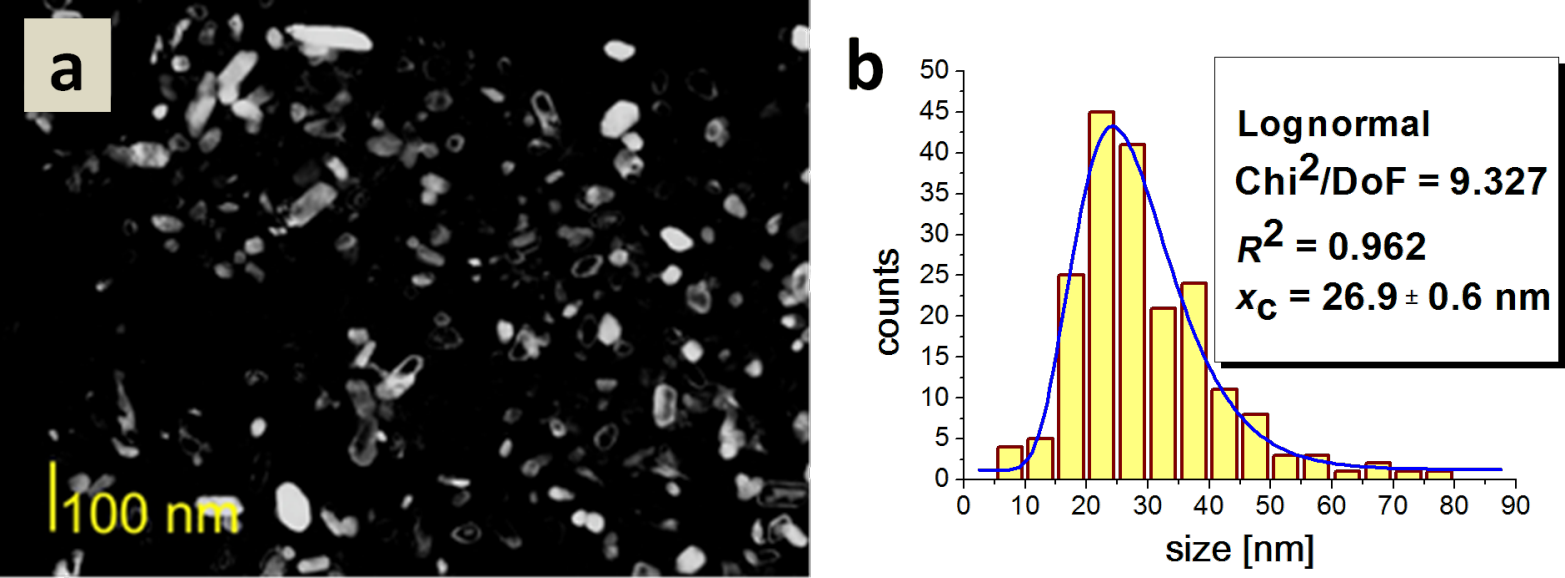
a) A dark field TEM image of Type 5 HAp; b) a histogram of the particle size distribution.

**Figure 11:**
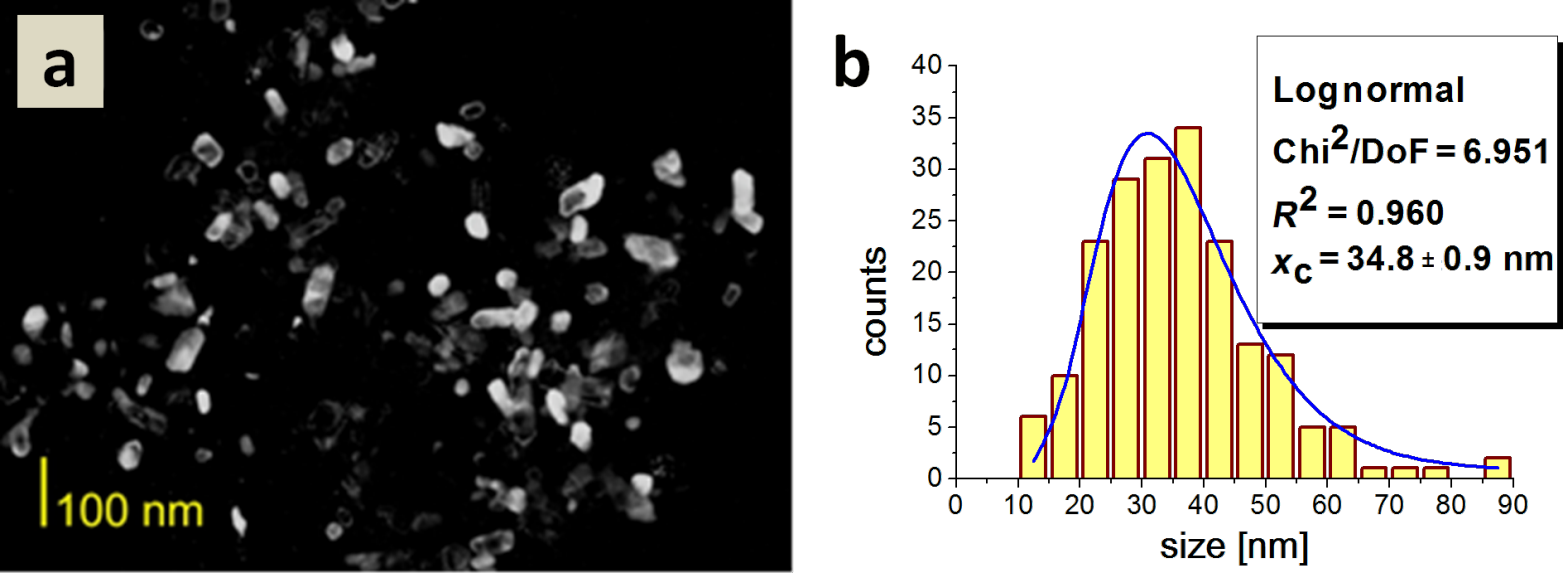
a) A dark field TEM image of Type 6 HAp; b) a histogram of the particle size distribution.

Hydroxyapatite nanoparticles are not spherical; they occur in the form of lamellas, which generates an error and discrepancies between the methods. When comparing the four methods used for determining the particle/crystallite size, the use of Scherrer’s equation yielded most accurate results [54]. It was the only method which could provide two dimensions (length and width) of the crystallites, based on which we inferred also the shape of the obtained particles (needles/lamellas). The same particle shape was independently confirmed by TEM measurements of HAp powders (see Figures 6–11 that follow), which confirms that Scherrer’s equation is capable of determining the shape of the particles. All the other methods of the Nanopowder XRD Processor Demo web application, converting the SSA results and TEM measurements using the dark field technique, provided the dimension (diameter) of particles/crystallites using the equations derived for spherical particles/crystallites. The adopted assumption is a significant simplification for the purpose of making calculations easier, which however introduces the systematic error in the results indicated in Table 5. However, the results of particle/crystallite size generated by all the mentioned methods constitute excellent data for controlling the quality and repeatability of HAp powders we obtain. Morphology was also evaluated by SEM and TEM techniques. Figure 5 presents SEM images of Type 1–Type 6 HAp nanoparticles.

The analysis of six hydroxyapatite types using SEM indicated that homogeneous powders of nanoscale particles were formed (Figure 5). It was observed that the morphology of the nanopowder changed from homogeneous, elongated particles (Type 1 and Type 2) to spherical particles (Type 3, 4, 5 and 6). This depended on the adopted synthesis parameters resulting from recrystallization. Crystallinity is the quantity of the crystalline phase in comparison with the content of the amorphous phase present in a material. HAp crystallization occurs in accordance with the Ostwald’s rule, so it usually forms from recrystallization of such precursor phases as octacalcium phosphate (OCP), dicalcium phosphate dihydrate (DCPD) and amorphous calcium phosphate (ACP) [55]. HAp crystallization during the synthesis ensues in accordance with the mechanism of crystallization from a solution. During the growth process, particles may grow on the surface of sediment or precipitate in all directions. Their growth may be expected to increase with the synthesis time until the crystallization process equilibrium has been achieved [55].

During the synthesis in the MSS2 reactor, HAp particles are dissolved and undergo recrystallization. Sharon Kehoe [50] noticed that when dealing with such a process, the smaller particles dissolve completely for the benefit of larger particles that continue to grow. As a result, the total number of particles decreases in line with the increase in their sizes, similar to the granularity of their area [50]. Both phenomena, the increase and decrease of roughness, result from the tendency to reduce the matter surface energy by a reduction of the SSA [31] (Table 4).

HAp morphology varies depending on the executed process parameters. The synthesis temperature growth (Type 3, 4, 5 and 6) leads to more regular, spherical HAp particles (Figure 5). Numerous reports have proved that synthesis at a low temperature leads to HAp crystals with needle morphology [31], which is confirmed by the SEM and TEM results of Type 1 HAp nanopowder. According to the literature review by Kehoe [50], many researchers who run synthesis at low temperatures obtained particles that were slightly thinner and longer, and more irregular, having less distinct borders. Additionally, these particles demonstrated a greater inclination to agglomerate [56]. It was also shown that the higher the crystallinity of the powder, the more regular the shape of the particle. This finding came from the observation that transition from irregular to regular morphologies, along with rise of the reaction temperature, coincided with the increase of the HAp particle crystallinity [50].

The TEM analysis indicated that each of the obtained hydroxyapatite types was a hydroxyapatite with P63/m space group, displaying a hexagonal structure with parameters *a* = 9.424 Å, *c* = 6.879 Å [57]. Variable synthesis parameters, such as time, temperature (pressure), or synthesis without the use of a reactor, were found to exert no visible impact on the phase composition and on the crystalline lattice parameters (Table 7). In all cases, obtaining hexagonal hydroxyapatite without the presence of foreign phases was observed.

A microscope image of Type 1 HAp (Figure 6), taken with the bright field technique, shows elongated structures with similar diameters of ≈6.5 nm (ranging from 4 to 9 nm), but with outliers ranging from more than ten to several hundred nanometers.

It was impossible to determine the length distribution of these structures precisely due to their agglomeration (Figure 6). The average nanoparticle size for Type 2 HAp was 7.3 nm (Figure 7).

An analysis of the histogram reveals that the sample contained a considerable fraction of similar sized particles, ranging from 5 to 10 nm. Crystallites did not display considerable differences in size and shape, but rather elongated, rice grain-shaped nanoparticles prevailed. The average particle size for Type 3 HAp was 11.7 nm. The sample contained a considerable fraction ranging from 5 to 20 nm. Type 3 HAp particles were of a similar size but quite diverse shape (Figure 8).

The average particle size for Type 4 HAp was 18.4 nm. The sample displayed a considerable diversity in average nanoparticle size in relation to samples of Type 1, 2 and 3 HAp. HAp crystallites for this type differed in shape and size as can be seen in Figure 9.

The average particle size for Type 5 HAp was 26.9 nm. The crystallites did not display significant differences in shape (Figure 10).

The average particle size for Type 6 HAp was 35 nm. Similarly, in this case, the particles did not display significant differences in shape, but they differed in size as shown in Figure 11.

The size distribution was found to be broader as compared with our previous publications [26–27]; this results primarily from an increased volume of the reaction mixture (6-fold increase in reagent volume). The volume of the reaction feedstock (350 mL, 3 kW) in the MSS2 reactor is considerably greater than that possible for the Ertec Magnum II reactor (70 mL, 0.6 kW). The increase in production scale (volume) contributes to decreased homogeneity of the obtained materials despite the use of microwave heating. It is common knowledge that scale-up in nanomaterial production is very problematic [58]. This is mainly due to the nonuniform heating of precursors. Despite the fact that we employ the microwave heating method [35,38,59], i.e., the most efficient and quickest method of supplying energy to reagents, an increase in the size distribution of the obtained particles in line with an increase in the volume of the reaction feedstock is noticeable. This results from restricted microwave penetration into the reaction chamber interior containing the water reaction suspension (to ≈2.5 cm). Another significant factor is the problem related to the stirring of the heated reagents during the microwave reaction, which determines the homogeneity of the obtained products. Precursor stirring during the synthesis in the MSS2 reactor is spontaneous and results from the temperature gradient in the reaction chamber. Stirring, phosphoric acid addition speed, pH of the synthesized suspension, precursor suspension stirring, and heating speed are some of the important factors taken into account when increasing the production scale of HAp nanoparticles.

As confirmed by the obtained results, fully crystalline HAp Type 1 was obtained without the use of the reactor at room temperature. The described reaction of obtaining HAp Type 1 is a reaction of the neutralization of Ca(OH)_2_ base with H_3_PO_4_ acid, where water is the sole byproduct. This is an exothermic process, the where the emitted energy is sufficient for the occurrence of the crystalline hydroxyapatite synthesis reaction. This is unique because according to Sharon Kehoe [50], ACP accompanied by small quantities of HAp was the main phase obtained from the synthesis at room temperature, whereas the quantity of HAp grew as the synthesis temperature increased [55]. The properties of the obtained primary product (HAp) are affected by the substrates used (Table 1) and the speed of adding (batching) acid to the calcium hydroxide suspension. Other works [60] have defined the optimum conditions for the synthesis of pure, monocrystalline HAp. According to the authors [60], the speed of adding the phosphoric acid solution should be moderate (below 100 mL/min), while the reaction temperature should be kept lower than 60 °C. Only then will the reaction ensue at 100% and the SSA of the obtained HAp will be ≈75 m^2^/g. If the speed of adding the phosphoric acid is very high, the pH value in the reaction environment considerably decreases. In this case, H_3_PO_4_ is not completely dissociated. An insufficient amount of phosphate ions bring about damage to the structure of HAp and the appearance of an undesirable phase, Ca(OH)_2_ [50]. In this paper, the adding speed was 1.2 mL/min, and the product obtained at room temperature was fully crystalline and characterized by a SSA as large as 258 m^2^/g. According to the authors of [55], the primary synthesis product at room temperature was ACP, and they obtained HAp only at 90 °C. This can be explained by the fact that the quantity of one of the reagents was greater than the quantity needed for producing HAp or that the speed of batching the reagents was greater than the speed of reaction of obtaining HAp at a given temperature. This resulting supersaturation of the suspension with calcium and phosphate ions was the primary reason for the formation of ACP [61].

**Figure 12:**
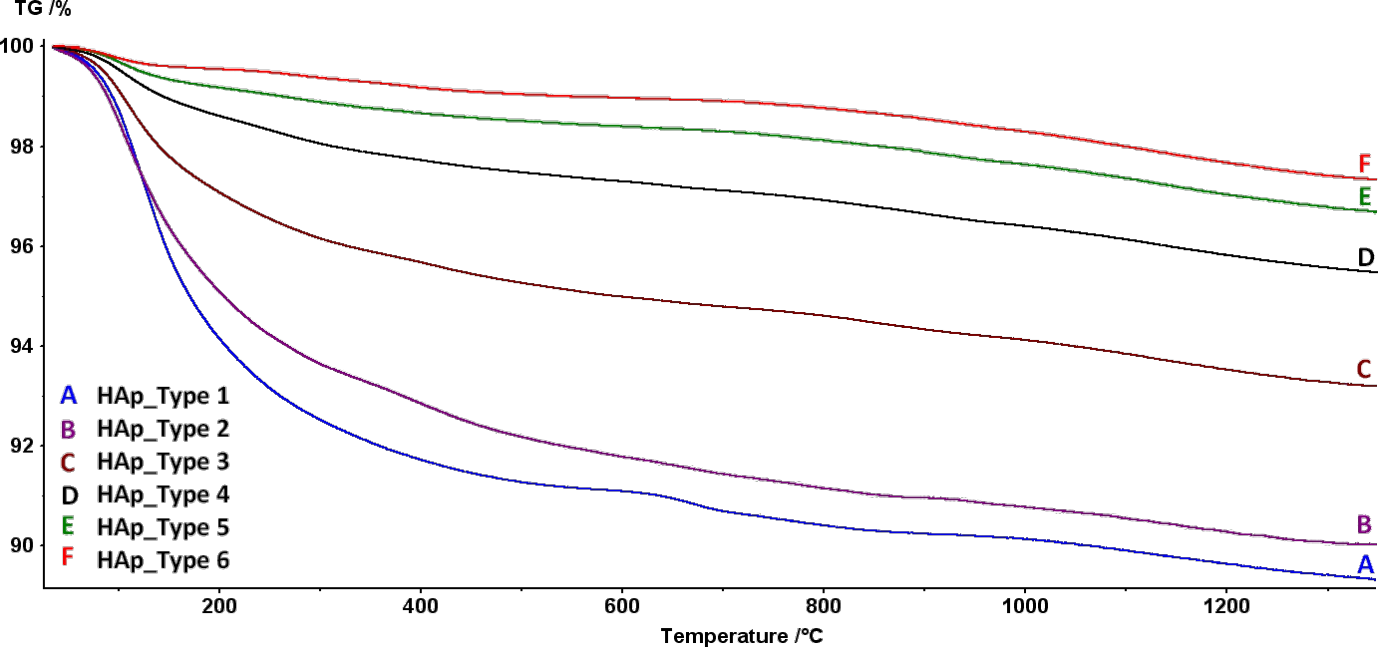
Results of thermogravimetric analysis for Type 1–Type 6 HAp nanopowder heated in helium atmosphere from room temperature to 1350 °C at 5 °C/min.

The thermogravimetric analysis (TGA) results (Table 8) provided information about total water content. The surface water and structural water can be distinguished. While heating, the water adsorbed on the surface evaporates up to 200 °C, then the structural water is evaporated subsequently in the temperature range 200–600 °C [62]. Even if this simplification is imprecise, it is sufficient to indicate trends in apatite hydration. Therefore, the total water content in our samples decreases in the following order: Type 1 HAp > Type 2 HAp > Type 3 HAp > Type 4 HAp > Type 5 HAp > Type 6 HAp (Figure 12).

**Table 8:** Analysis of the quantity of water adsorbed on the surface and present in the structure of the tested hydroxyapatites.

Sample	SSA, *a*_s_ (m^2^/g)	Weight loss 25–200 °C (absorbed water), (%)	Weight loss above 200 °C (lattice water), (%)	Total weight loss up to 1350 °C, (%)

HAp Type 1	258	5.85	4.83	10.68
HAp Type 2	211	4.91	5.08	9.99
HAp Type 3	149	2.93	3.88	6.81
HAp Type 4	85	1.40	3.13	4.53
HAp Type 5	61	0.84	2.48	3.32
HAp Type 6	51	0.46	2.22	2.68

Thermogravimetric tests reveal that the greatest quantity of water adsorbed on the particle surface was measured in the case of Type 1 and 2 HAp nanopowder, which is probably connected with the highly developed SSA. HAp is a hygroscopic material and hence the primary component adsorbed on the surface is water. The remaining analyzed powders are characterized by a lower SSA value, so the quantity of adsorbed water is accordingly lower. Powders 1 and 2 are also characterized by the highest content of structural water, i.e., built in the crystalline lattice (200–600 °C).

## Conclusion

A synthesis method was developed allowing for HAp nanoparticles to be produced with controlled size ranging from 8 to 39 nm. The synthesis was carried out using two methods: precipitation method with the precursor Ca(OH)_2_ + H_3_PO_4_, where the single by-product was water and microwave hydrothermal synthesis, where the Type 1 HAp suspension was used as the precursor and heated in the MSS2 microwave reactor. The test proved that fully crystalline, clear nonstoichiometric, nanoscale HAp can be obtained without the use of the MSS2 reactor, which was a novelty.

The average HAp size was controlled by synthesis conditions such as time, temperature, and pressure. Crystalline HAp with hexagonal structure with pure phase was obtained. The HAp nanoparticles were characterized by a homogeneous morphology with needle or spherical/elliptical shape and a significant specific surface area. The morphology and density of the nanoparticles was found to depend on their size. The reaction time was short and the process was carried out entirely in water. MSS2 technology uses high power density, microwave radiation and enables a nanoscale, fully crystalline structure to be produced for all types of hydroxyapatite. The MSS2 reactor permits the control of the parameters of the obtained hydroxyapatite nanopowder, for example, the surface area in the range 51–258 m^2^/g, and density of 2.86–3.04 g/cm^3^. The developed precipitation and MHS methods enabled homogeneous HAp nanoparticles to be obtained with controlled and narrow particle size distribution. However, when increasing the production scale, the size distribution of HAp NPs was increased. An advantage of the developed precipitation synthesis and MSH methods is the low cost of the whole process and the purity of the obtained materials. The XRD results confirmed that Type 1 and Type 2 hydroxyapatite is a synthetic equivalent of natural apatite. A considerable content of structural water and water adsorbed in nanoscale HAp of diameter less than 20 nm, reaching 10 wt %, was found.

The unique design of the MSS2 rector permits precise synthesis time control with an accuracy of 1 s. One of the MSS2 reactor’s operation features is the emptying the reactor chamber immediately after the heating has ended, which leads to rapid cool down and freezing of the reaction, whereby highest purity preserved. With the MSS2 reactor, it is possible to obtain intermediates of any reaction at any time during the microwave synthesis without post-reaction reheating effects. Microwave irradiation is a highly efficient heating method for transferring energy into the reaction chamber and provides more uniform heating with a rapid temperature rise in comparison with conventional heat transfer methods. MHS is generally much simpler, cleaner, faster, very energy efficient and more economical than conventional methods. This low-energy technology is also environmentally friendly.

The technology is ready to be employed in production conditions, while powder parameters can be adjusted to the consumer needs. The method of nanoparticle size control developed herein can be used for optimizing the HAp properties in specific applications.
